# Epidemiological Characteristics of Respiratory Syncytial Virus Infection Among Hospitalized Children With Acute Respiratory Tract Infections From 2014 to 2022 in a Hospital in Hubei Province, China: Longitudinal Surveillance Study

**DOI:** 10.2196/43941

**Published:** 2023-04-27

**Authors:** Xing-Wen Hu, Yiguo Zhou, Song Yi, Wan-Xue Zhang, Xin-Rui Wang, Juan Du, Qing-Bin Lu

**Affiliations:** 1 Department of Clinical Laboratory Maternal and Child Health Hospital of Hubei Province Wuhan China; 2 Department of Health Policy and Management School of Public Health Peking University Beijing China; 3 Department of Medical Genetic Center Maternal and Child Health Hospital of Hubei Province Wuhan China; 4 Department of Epidemiology and Biostatistics School of Public Health Peking University Beijing China; 5 Department of Laboratorial Science and Technology & Vaccine Research Center School of Public Health Peking University Beijing China; 6 Global Center for Infectious Disease and Policy Research & Global Health and Infectious Diseases Group Peking University Beijing China; 7 Key Laboratory of Epidemiology of Major Diseases (Peking University) Ministry of Education Beijing China

**Keywords:** respiratory syncytial virus, acute respiratory tract infection, epidemiological characteristics, China, COVID-19

## Abstract

**Background:**

Longitudinal studies characterizing the epidemic trend of respiratory syncytial virus (RSV) in Hubei Province are scarce.

**Objective:**

We aimed to depict the dynamics of the RSV epidemic among hospitalized children with acute respiratory tract infections (ARTIs) during 2014 to 2022 in the Maternal and Child Health Hospital of Hubei Province and investigate the influence of the 2-child policy and the COVID-19 pandemic on RSV prevalence.

**Methods:**

The medical records and testing results of hospitalized children with ARTI from January 2014 to June 2022 were extracted. Nasopharyngeal samples were tested with direct immunofluorescence assay. Detection rates of RSV were categorized according to the diagnosis of patients: (1) overall, (2) upper respiratory tract infection (URTI), and (3) lower respiratory tract infection (LRTI). Poisson regression models were used to investigate the association between RSV detection rate and age, gender, or diagnosis. The detection rates of RSV before and after the implementation of the universal 2-child policy were compared using a Poisson regression model. Multiple comparisons of RSV detection rates were conducted among 3 stages of the COVID-19 pandemic using chi-square tests. Seasonal autoregressive integrated moving average was performed to predict RSV behaviors from February 2020 to June 2020 under the assumption of a non-COVID-19 scenario.

**Results:**

Among 75,128 hospitalized children with ARTI, 11.1% (8336/75,128) were RSV-positive. Children aged <1 year had higher detection rates than older children (4204/26,498, 15.9% vs 74/5504, 1.3%; *P*<.001), and children with LRTI had higher detection rates than children with URTI (7733/53,145, 14.6% vs 603/21,983, 2.7%; *P*<.001). Among all the children, a clear seasonal pattern of the RSV epidemic was observed before 2021. Most of the highest detection rates were concentrated between December and February. The yearly detection rate of RSV remained at a relatively low level (about 8%) from 2014 to 2017, then increased to 12% and above from 2018. The highest monthly detection rate was in December 2018 (539/1493, 36.1%), and the highest yearly rate was in 2021 (1372/9328, 14.7%). There was a moderate increase in the RSV detection rate after the 2-child policy was implemented (before: 860/10,446, 8.2% vs after: 4920/43,916, 11.2%; *P*<.001). The largest increase, by 5.83%, occurred in children aged <1 year. The RSV epidemic level decreased sharply in the short term after the COVID-19 outbreak (detection rate before: 1600/17,010, 9.4% vs after: 32/1135, 2.8%; *P*<.001). The largest decrease, by 12.0%, occurred in children aged <1 year, but a rebounding epidemic occurred after 2020 (680/5744, 11.8%; *P*<.001).

**Conclusions:**

Children have been experiencing increased prevalence of RSV since 2018 based on surveillance from a hospital in Hubei Province with a large sample size. The 2-child policy might have increased the RSV prevalence, and the COVID-19 epidemic had a temporary inhibitory effect on RSV transmission. Vaccines against RSV are urgently needed.

## Introduction

Respiratory syncytial virus (RSV) is an enveloped, single-stranded, negative-strand RNA virus belonging to the genus *Orthopneumovirus* within the family *Pneumoviridae* [1]. RSV is one of the most common pathogens leading to acute lower respiratory tract infections (LRTIs) among infants [2]. It has been reported that approximately 70% of children are infected in the first year after birth, and almost 100% are infected in their first 2 years [3]. In adults and older children, RSV usually causes mild clinical cold-like signs and symptoms such as congested or runny nose, cough, fever, sore throat, sneezing, and headache, which can disappear in 1 week to 2 weeks [4]. In contrast, RSV can cause much more severe outcomes including pneumonia, bronchiolitis, and even death in young children, especially in infants with underlying diseases or a weak immune system [4]. Recent research estimated that 33.0 million episodes, 3.6 million hospital admissions, 26,300 in-hospital deaths, and 101,400 overall deaths were attributed to RSV-associated acute LRTI in children under 5 years old in 2019 globally [5], and more than 95% of episodes and 97% of deaths happened in low and middle-income countries. RSV-attributable deaths were responsible for 1 in 50 and 1 in 28 deaths in children aged under 5 years old and children aged 28 days to 6 months, respectively. In addition, RSV infection in early life can cause an increased risk of multiple long-term adverse effects including asthma and decreased lung function [6]. RSV infection can also lower immunity and contribute to the risk of COVID-19 and the severity of COVID-19 illnesses [4]. Nevertheless, no drug has been successfully developed to specifically treat RSV-related illnesses to date.

Similar to other respiratory viruses, RSV is spread easily on infected respiratory droplets by air to people’s eyes, noses, and mouths if people with RSV cough or sneeze near susceptible individuals. Common measures to prevent RSV infection include washing hands frequently, limiting children’s contact with people who have cold-like symptoms, keeping a hygienic environment, and not sharing drinking glasses with others [4]. Dozens of promising vaccine candidates against RSV based on multiple technologies are being developed; however, no candidate has yet completed a phase 3 trial [1]. Some of these vaccine candidates may receive regulatory approval in the near future [1].

Previous studies revealed that the RSV epidemic has obvious seasonal patterns worldwide [7-9]. In the northern hemisphere, it usually occurs from October/November to April/May, while it occurs from May to September in the southern hemisphere [10]. In light of the regionality and seasonality of the epidemic, investigation of conducting continuous surveillance of RSV activities widely in different regions is warranted, especially in metropolitan cities with larger population movement, to enable early warning and medical preparations. During the early stage of the COVID-19 pandemic, marked decreases in non-COVID-19 respiratory infections including RSV that were observed in previous epidemic seasons as multiple mitigation measures were adopted to cope with COVID-19 [11-17]. The strictest nonpharmaceutical interventions (NPIs) including city lockdown, social distancing, nucleic acid testing, wearing masks, and suspension of work and school were implemented in Wuhan to contain the COVID-19 outbreak in the first half of 2020. After the control of the first wave of COVID-19, most NPIs were lifted or relaxed, and nucleic acid testing was regarded as the leading component of measures to control COVID-19. It was a typical example of assessing the impact of interventions against COVID-19 on the epidemic regularity of RSV. In addition, the universal 2-child policy was implemented in Hubei Province in 2016 and lifted the 1-child limit and allowed all couples, regardless of residence in urban or rural areas and regions and ethnic groups, to have 2 children. The potential increased number of newborns in Hubei Province after the policy shift might have influenced the epidemiological characteristics of diseases in young children. Based on data from the largest maternal and child health hospital in Hubei Province of China from 2014 to 2022, we aimed to depict the dynamics of the RSV epidemic in the past 8 years and investigate the influence of the 2-child policy and the COVID-19 pandemic on RSV prevalence.

## Methods

### Design, Setting, and Population

This study retrospectively reviewed RSV detection results among hospitalized children with acute respiratory tract infection (ARTI) from January 2014 to June 2022 in the Maternal and Child Health Hospital of Hubei Province. The Maternal and Child Health Hospital of Hubei Province is the largest tertiary hospital specializing in maternal and child health service and academic research in Hubei Province, with 1900 inpatient beds and more than 2.77 million outpatient visits in 2019 [18]. Hospitalized children were included if they met the following criteria: (1) diagnosed with ARTI and (2) aged less than 18 years. Specifically, ARTI consisted of upper respiratory tract infection (URTI) and LRTI. LRTI included bronchiolitis and pneumonia [19]. Children were divided into 4 age groups: <1 year, 1-2 years, 3-5 years, and 3-17 years. Basic information about the patients including demographics, symptoms, diagnosis, and examination results was extracted from the hospital case information database.

### Samples and Laboratory Testing

Respiratory tract samples were collected within 24 hours after admission using nasopharyngeal swabs, which were stored in centrifuge tubes containing 3 mL saline. RSV was detected through direct immunofluorescence assay with a D^3^ Ultra DFA Respiratory Virus Screening and ID Kit (Diagnostic Hybrids Inc). Detailed laboratory operations under the guidance of the kit instructions were as follows: (1) fluid containing samples was blended fully using a vortex oscillator; (2) tubes were centrifuged at 500×g for 10 minutes, then only 150 μL supernatant and sediment were kept; (3) supernatant and sediment were mixed for cell suspension, and 150 μL cell suspension was added to a glass slide, dried, immersed in cold acetone solution for 10 minutes, and dried again; (4) a drop of fluorescein isothiocyanate–labeled monoclonal antibody (about 25 μL) was added to the glass slide to cover the sample and incubated for 30 minutes in a wet box at 37 °C and rinsed and covered by a glass cover; (5) the glass slide containing samples was observed under a fluorescent microscope (Olympus BX53). A sample was regarded as positive for RSV when at least 2 positive cells with apple green fluorescence were observed in the field of view at a 200-fold magnification of the microscope.

### Statistical Analysis

The detection rate was calculated as the number of RSV-positive samples divided by the total number of samples, with the 95% CI of the detection rate determined using the Clopper-Pearson method. In all analyses, detection rates are presented in 3 categories according to the diagnosis of patients: overall, URTI, and LRTI. In each category, detection rates were further described according to age group and gender. An adjusted risk ratio (RR) and 95% CI were obtained using a Poisson regression model to show the relative risk of getting RSV at 1 level compared with the reference level in 1 variable, after adjusting for the other 2 variables. For example, the risk of RSV infection in male children versus female children was assessed by adjusting the impact of age and diagnosis. Line plots and bar plots were generated to delineate the detection rate and the number of samples, respectively. A heat map was generated to compare the seasonal distribution of the RSV epidemic across different years, in which a detection rate of 8% was assumed to be an RSV epidemic. Considering the 2-child policy was implemented in 2016 in Hubei Province, the difference in the pooled RSV detection rates between the 2014-2015 and 2017-2019 periods was illustrated by calculating an adjusted RR in the Poisson regression model. These statistical comparisons were considered significant at *P*<.05. According to the COVID-19 status in Hubei Province, 3 stages were determined: stage 1 (prior to the COVID-19 pandemic: February to June in each year from 2017 to 2019), stage 2 (early COVID-19 outbreak: February 2020 to June 2020), and stage 3 (COVID-19 normalization period: February to June in each year from 2021 and 2022). The change in the RSV epidemic in the 2 periods after the COVID-19 outbreak in contrast to the period prior to COVID-19 was investigated. The chi-square test was used in multiple comparisons with statistical significance set at *P*<.025 in the Bonferroni method. Seasonal autoregressive integrated moving average (SARIMA) was used to present the difference between the predicted epidemic level of RSV from February 2020 to June 2020 in a non-COVID-19 scenario and the true detection rate of RSV in Hubei Province. Time series data of URTI cases were not analyzed with a SARIMA model as they were not stationary in the unit root test. Microsoft Excel, Stata 17 (StataCorp), and R 4.1.2 (R Core Team) were used for data processing and visualization.

### Ethical Considerations

This study was approved by the clinical research ethics committee of the Maternal and Child Health Hospital of Hubei Province (2022IEC052). Informed consent was exempted due to the retrospective nature of our study. Data used in this study were anonymized and deidentified.

## Results

### Basic Characteristics of the Overall Sample

A total of 75,128 children from January 2014 to June 2022 met the inclusion criteria of this study (Table 1). These participants primarily consisted of children aged <2 years (50,417/75,128, 67.1%), who were male (44,959/75,128, 59.8%), and who were diagnosed with LRTI (53,145/75,128, 70.7%). Among all the children, 11.1% (8336/75,128) were RSV-positive. The children aged <1 year had the highest RSV detection rate (4204/26,498, 15.9%), followed by children aged 1 year to 2 years (2777/23,919, 11.6%), and rates in both age groups were significantly higher than that for older children (*P*<.001). No significant difference in the RSV detection rate existed between male children and female children (*P*=.54). In addition, the children with LRTI were more likely to be RSV-positive than those with URTI (7773/53,145, 14.6% vs 603/21,983, 2.7%; adjusted RR 4.88, 95% CI 4.50-5.30; *P*<.001). Among 986 children requiring care in the intensive care unit, 256 (26.0%) were positive for RSV.

**Table 1:** Basic characteristics of the overall sample (n=75,128).

Variables		Positive tests, n	Tests, n	Detection rate, % (95% CI)	Adjusted RR^a^ (95% CI)	*P* value	
Overall		8336	75,128	11.1 (10.9-11.3)	—^b^	—	
**Age (years)**							
	<1	4204	26,498	15.9 (15.4-16.3)	9.84 (7.83-12.36)	<.001^c^	
	1-2	2777	23,919	11.6 (11.2–12.0)	8.15 (6.48-10.25)	<.001^c^	
	3-5	1281	19,207	6.7 (6.3-7.0)	4.61 (3.65-5.82)	<.001^c^	
	6-17	74	5504	1.3 (1.1-1.7)	Reference		
**Gender**							.54^d^
	Male	5123	44,959	11.4 (11.1-11.7)	0.99 (0.95-1.03)		
	Female	3213	30,169	10.7 (10.3-11.0)	Reference		
**Diagnosis**							<.001^e^
	URTI^f^	603	21,983	2.7 (2.5-3.0)	Reference		
	LRTI^g^	7733	53,145	14.6 (14.3-14.9)	4.88 (4.50-5.30)		

^a^RR: risk ratio.

^b^Not calculable in the overall population.

^c^Adjusted for gender and diagnosis.

^d^Adjusted for age and diagnosis.

^e^Adjusted for age and gender.

^f^URTI: upper respiratory tract infection.

^g^LRTI: lower respiratory tract infection.

### Time Trend and Seasonal Pattern of RSV

**Figure 1:**
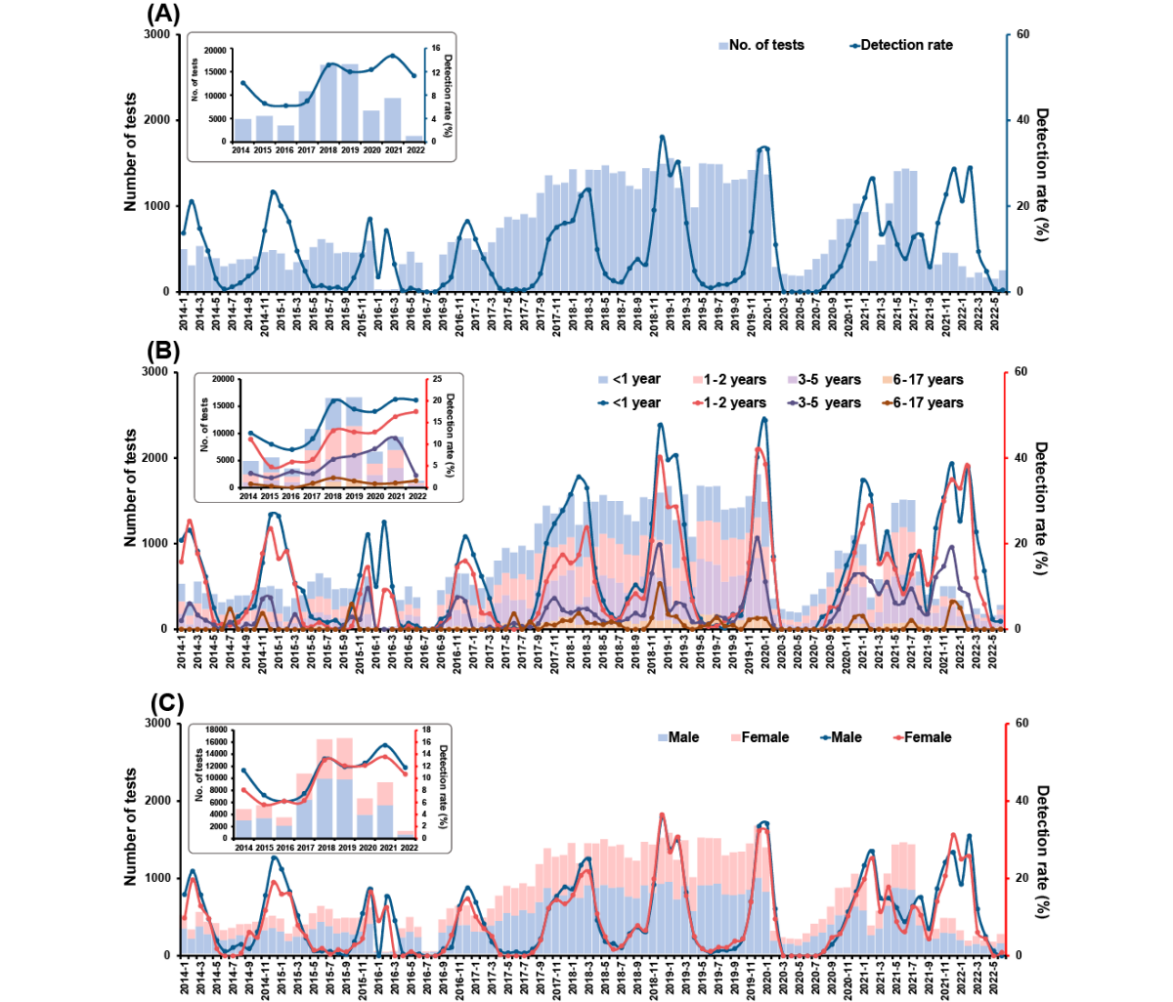
Monthly distribution of tests for (bars) and detection rate of (lines) respiratory syncytial virus from 75,128 hospitalized children with acute respiratory tract infection during 2014 to 2022: (A) overall sample, (B) by age group, (C) by gender.

Among all the children, a clear seasonal pattern of the RSV epidemic was observed before 2021. Most top detection rates were concentrated between December and February, except for March in 2018 (Figure 1). The yearly detection rate of RSV remained at a relatively low level (about 8%) from 2014 to 2017, then increased to 12% and above from 2018. The highest monthly detection rate was in December 2018 (539/1493, 36.1%), and the highest yearly rate was in 2021 (1372/9328, 14.7%). Notably, from May to September in the years before 2021, the RSV detection rate was maintained at an extremely low level, close to zero, while a wave of a moderate epidemic (about 10%) arose in the same period in 2021. From the perspective of age, obvious differences in the RSV detection rate across 3 age groups were seen. Younger children had a higher RSV detection rate. However, RSV detection rates were hard to distinguish between children <1 year old and children 1 year to 2 years old after January 2021. The detection rates between the male and female children almost coincided.

**Figure 2:**
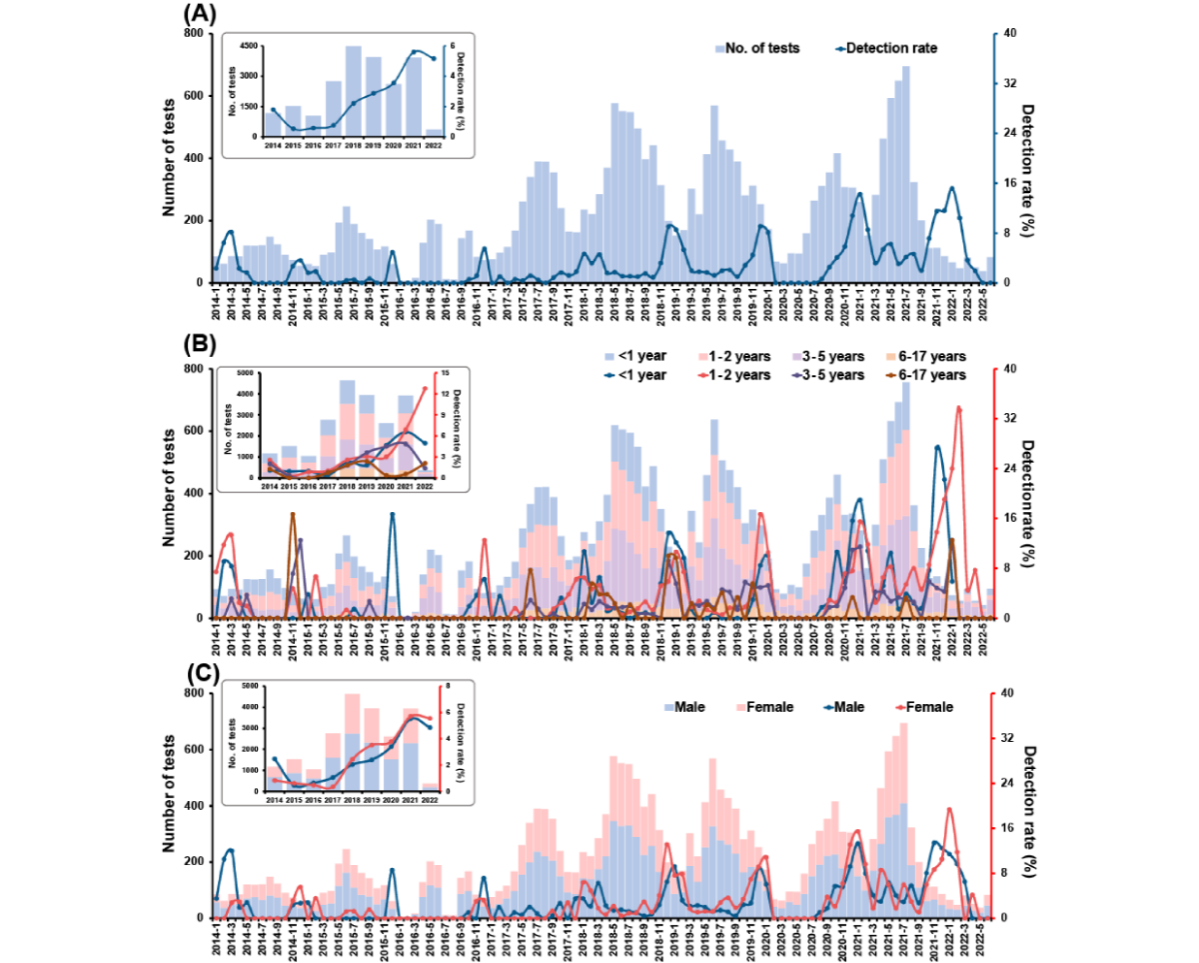
Monthly distribution of tests for (bars) and detection rates of (lines) respiratory syncytial virus from 21,983 hospitalized children with acute upper respiratory tract infection during 2014 to 2022: (A) overall sample, (B) by age group, (C) by gender.

**Figure 3:**
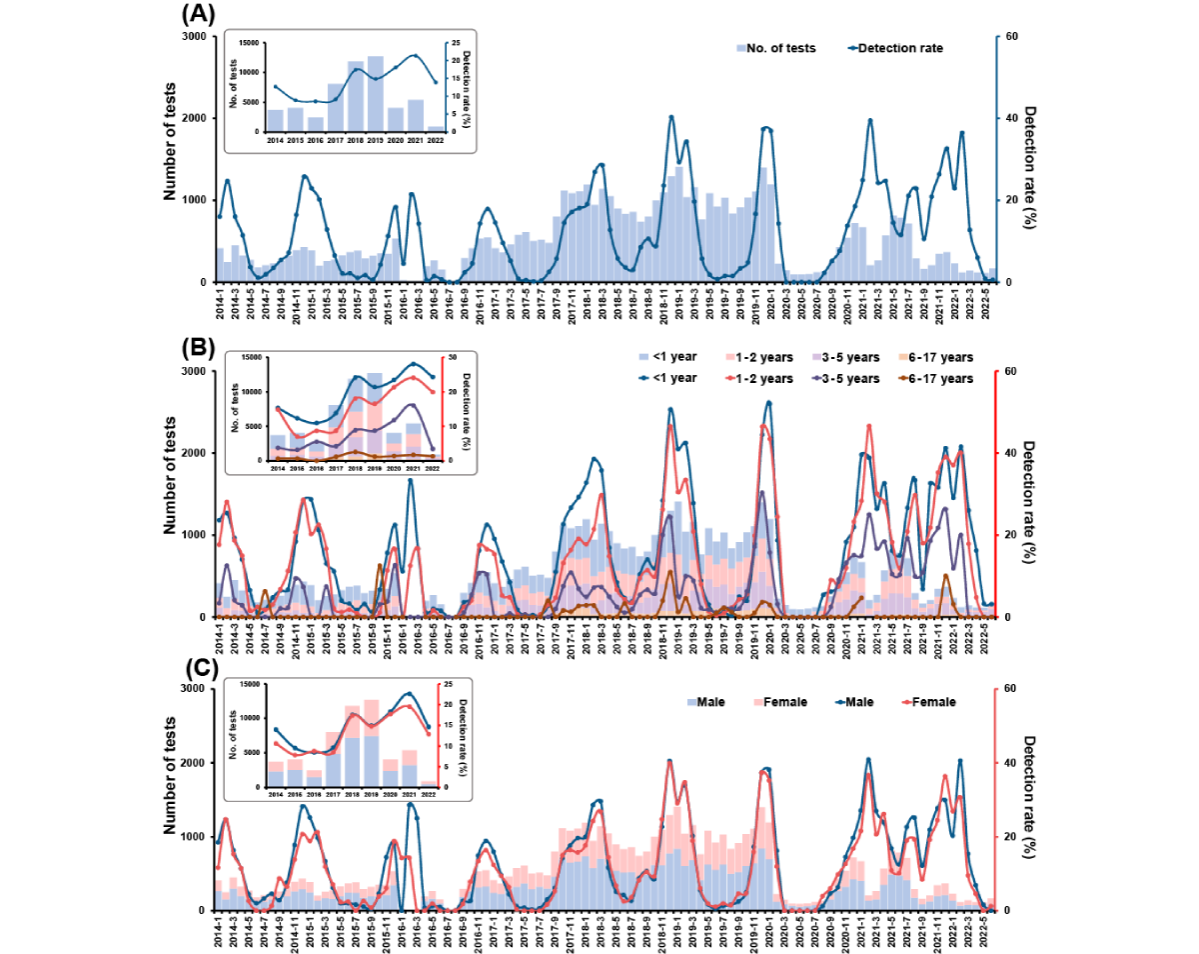
Monthly distribution of tests for (bars) and detection rate of (lines) respiratory syncytial virus from 53,145 hospitalized children with acute lower respiratory tract infection during 2014 to 2022: (A) overall sample, (B) by age group, (C) by gender.

Among the children with URTI, a seasonal peak also existed in December or January (Figure 2). Monthly detection rates of more than 10% were only seen in 2021 and 2022. From 2015 to 2021, the yearly RSV detection rate in URTI cases increased continuously from 0.5% (8/1521) to 5.6% (220/3929). Children aged 1 year to 2 years had a higher positive RSV detection rate than those aged 6 years to 17 years (258/8017, 3.2% vs 26/2136, 1.2%, *P*<.001; Table S1 in Multimedia Appendix 1). No significant difference in RSV detection rate was found between genders (*P*=.37). Among the children with LRTI, the trend and seasonal pattern of RSV detection rate were similar to that in all the children (Figure 3). The monthly RSV detection rate peaked in December 2018 (521/1294, 40.3%), and the yearly detection rate peaked in 2021 (1152/5399, 21.3%). Children aged <1 year (4055//20,849, 19.5%) or 1 year to 2 years (2519/15,902, 15.8%) had obviously higher RSV-positive rates than the oldest age group (48/3368, 1.4%; all *P*<.001; Table S2 in Multimedia Appendix 1). No difference was observed between genders (*P*=.57).

**Figure 4:**
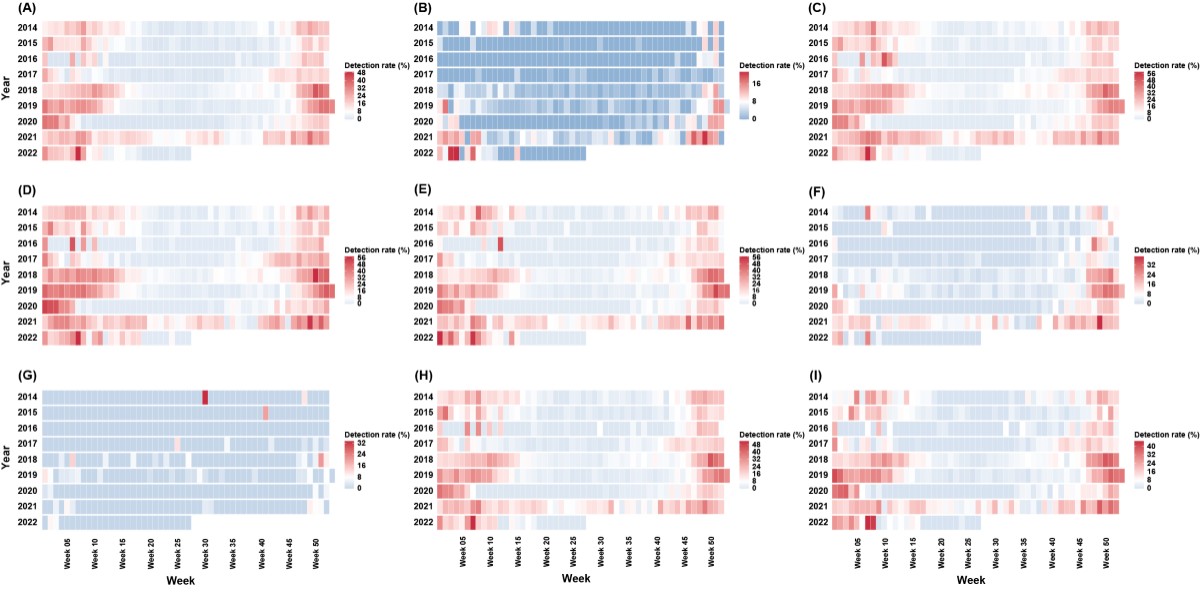
Heat maps of the respiratory syncytial virus detection rate by week during 2014 to 2022: (A) overall sample, (B) in those with upper respiratory tract infection, (C) in those with lower respiratory tract infection, (D) in those <1 year old, (E) in those 1 year to 2 years old, (F) in those 3 years to 5 years old, (G) in those 6 years to 17 years old, (H) in male children, (I) in female children.

The heat maps show the beginning and end weeks of the RSV epidemic in each year (Figure 4 and Multimedia Appendix 2). A similar distribution of the RSV epidemic was observed between all the children and those with LRTI. The earliest beginning of the epidemic (about the 40th week) happened in 2017 and 2021, and the latest end of the epidemic (the 15th and 20th weeks) happened in 2018 and 2021, respectively. Particularly in 2021, RSV had the longest epidemic period lasting 40 weeks (about 77% of the whole year). In contrast, the shortest epidemic period of only 15 weeks occurred in 2020. Things were quite different in children with URTI. The RSV epidemic lasted a short number of weeks (<5 weeks) in the years before 2020. However, a long RSV epidemic period occurred in 2021 (16 weeks). Heat maps of the 2 younger age groups and the 2 genders did not provide different information from that of all the children. A much shorter epidemic period was seen for children aged 3 years to 5 years or 6 years to 17 years.

### Impact of the 2-Child Policy on the RSV Epidemic

Among all the children, there was a moderate increase in the RSV detection rate in most subgroups, except for children aged 6 years to 17 years, after the 2-child policy was implemented (all *P*<.001; Table 2). The largest increase, by 5.83%, occurred in children aged <1 year. Children aged 6 years to 17 years had the highest risk of being infected after the implementation of the policy (adjusted RR 2.80, 95% CI 0.88-8.90). Similar results were found with children with LRTI. Among the children with URTI, the increased RSV detection rates in only the entire group of children and female children were significant (*P*=.001 and *P*=.002, respectively). Though female children had a high risk of RSV infection after the implementation of the 2-child policy (adjusted RR 3.12, 95% CI 1.52-6.39), the prevalence of RSV increased slightly from 0.7% (8/1133) to 2.3% (107/4658).

**Table 2:** Comparison of the detection rate of respiratory syncytial virus before and after the implementation of the universal 2-child policy.

Group				Before (2014-2015)							After (2017-2019)							Adjusted RR^a^ (95% CI)			*P* value
				Positive tests		Number of tests		Detection rate, % (95% CI)		Positive tests			Number of tests		Detection rate, % (95% CI)						
Total population				860		10,446		8.23 (7.71-8.78)		4920			43,916		11.20 (10.91-11.50)		1.52 (1.42-1.63)			<.001^b^	
**Total population**																					
	**Age (years)**																				
		<1	573		5105		11.22 (10.37-12.12)		2554			14,977		17.05 (16.45-17.66)		1.50 (1.38-1.63)			<.001^c^		
		1-2	234		2984		7.84 (6.9-8.87)		1626			14,248		11.41 (10.89-11.95)		1.41 (1.24-1.60)			<.001^c^		
		3-5	50		1850		2.70 (2.05-3.54)		683			11,224		6.09 (5.66-6.55)		2.26 (1.70-2.99)			<.001^c^		
		6-17	3		507		0.59 (0.20-1.72)		57			3467		1.64 (1.27-2.12)		2.80 (0.88-8.90)			.08^c^		
	**Gender**																				
		Male	589		6436		9.15 (8.46-9.88)		2966			26,183		11.33 (10.95-11.72)		1.41 (1.30-1.53)			<.001^d^		
		Female	271		4010		6.76 (6-7.58)		1954			17,733		11.02 (10.56-11.49)		1.77 (1.57-1.99)			<.001^d^		
URTI^e^				29		2691		1.08 (0.72-1.54)		236			11,340		2.08 (1.83-2.36)		1.88 (1.28-2.76)			.001^f^	
**URTI**																					
	**Age (years)**																				
		<1	10		1001		1.00 (0.48-1.83)		44			2713		1.62 (1.18-2.17)		1.62 (0.82-3.21)			.17^g^		
		1-2	13		956		1.36 (0.73-2.31)		99			4164		2.38 (1.94-2.89)		1.75 (0.98-3.11)			.06^g^		
		3-5	5		536		0.93 (0.40-2.16)		72			3282		2.19 (1.74-2.75)		2.36 (0.96-5.81)			.06^g^		
		6-17	1		198		0.51 (0.09-2.81)		21			1181		1.78 (1.17-2.71)		3.53 (0.48-25.80)			.22^g^		
	**Gender**																				
		Male	21		1558		1.35 (0.84–2.05)		129			6682		1.93 (1.61-2.29)		1.41 (0.89-2.23)			.15^h^		
		Female	8		1133		0.71 (0.31–1.39)		107			4658		2.30 (1.89-2.77)		3.12 (1.52-6.39)			.002^h^		
LRTI^i^				831		7755		10.72 (10.04-11.43)		4684			32,576		14.38 (14-14.76)		1.51 (1.41-1.62)			<.001^f^	
**LRTI**																					
	**Age (years)**																				
		<1	563		4104		13.72 (12.68-14.81)		2510			12,264		20.47 (19.76-21.19)		1.49 (1.37-1.63)			<.001^g^		
		1-2	221		2028		10.90 (9.57-12.34)		1527			10,084		15.14 (14.45-15.86)		1.39 (1.22-1.59)			<.001^g^		
		3-5	45		1314		3.42 (2.57-4.55)		611			7942		7.69 (7.12-8.30)		2.24 (1.67-3.02)			<.001^g^		
		6-17	2		309		0.65 (0.18-2.33)		36			2286		1.57 (1.14-2.17)		2.43 (0.59-10.04)			.22^g^		
	**Gender**																				
		Male	568		4878		11.64 (10.76-12.58)		2837			19,501		14.55 (14.06-15.05)		1.41 (1.30-1.53)			<.001^h^		
		Female	263		2877		9.14 (8.11-10.25)		1847			13,075		14.13 (13.53-14.74)		1.72 (1.52-1.95)			<.001^h^		

^a^RR: rate ratio.

^b^Adjusted for age, gender, and diagnosis.

^c^Adjusted for gender and diagnosis.

^d^Adjusted for age and diagnosis.

^e^URTI: upper respiratory tract infection.

^f^Adjusted for age and gender.

^g^Adjusted for gender.

^h^Adjusted for age.

^i^LRTI: lower respiratory tract infection.

### Impact of the COVID-19 Outbreak on the RSV Epidemic

**Figure 5:**
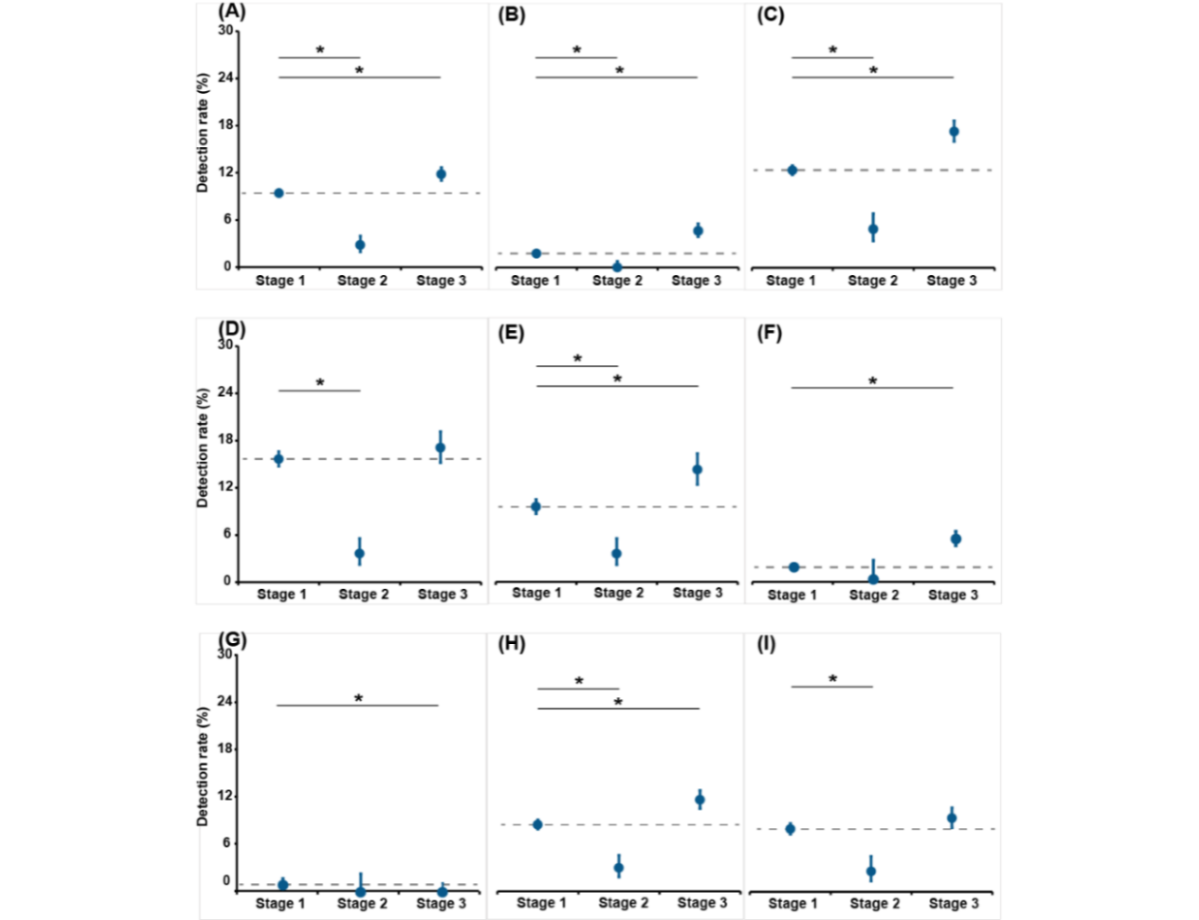
Comparison of the respiratory syncytial virus detection rate among periods according to COVID-19 epidemic status (stage 1: February to June in each of 2017, 2018, and 2019; stage 2: February 2020 to June 2020; stage 3: February to June in each of 2021 and 2022): (A) overall sample, (B) those with upper respiratory tract infection, (C) those with lower respiratory tract infection, (D) in those <1 year old, (E) in those 1 year to 2 years old, (F) in those 3 years to 5 years old, (G) in those 6 years to 17 years old, (H) in male children, (I) in female children. *P<.05.

Among all the children, compared with the pooled levels from February to June in each year from 2017 to 2019, all the stratifications except for the 2 older groups achieved significant reductions in the RSV detection rate during the earliest months of the COVID-19 pandemic (February 2020 to June 2020; Figure 5, Multimedia Appendix 3, and Table S3 in Multimedia Appendix 1). Of these, the sharpest decline, by 12.0%, occurred in children aged <1 year. Among the children with URTI, only the overall sample and male children showed declines of statistical significance. Among the children with LRTI, only the children aged 1 year to 2 years, 3 years to 5 years, or 6 years to 17 years did not have significantly decreased RSV detection rates. The largest decrease (13.8%) was observed in children aged <1 year.

Among all the children, compared with the pooled levels during February to June in each of 2017 to 2019, the overall RSV detection rate increased from 9.4% (1600/17,010) to 11.8% (680/5744) during the same periods in 2021 and 2022 (Figure 5, Multimedia Appendix 3, and Table S3 in Multimedia Appendix 1). All the stratifications, except for children aged <1 year and female children, had significant increases in the RSV detection rate. Of these, children aged 1 year to 2 years had the largest increase, by 4.7%. Among the children with URTI, all the stratifications, except for children aged 6 years to 17 years, had significant increases. The children aged 1 year to 2 years had the largest increase, by 4.3%. Among the children with LRTI, all the stratifications, except for children aged 6 years to 17 years, had significant increases, with the largest increase in children aged 1 year to 2 years, by 7.7%.

Multimedia Appendix 4 compares the observed rates with the predicted rates of RSV detection from February 2020 to June 2020 based on the SARIMA model. Among all the children, the forecasted RSV detection rates were much higher than the actual values. The largest decrease, by 28.5%, in the RSV detection rate happened in March 2020, denoting the most significant impact of the COVID-19 outbreak. In all the stratified analyses, the observed rates were significantly lower than the lower limit of the 95% CI of the forecasted rates representing the counterfactual scenario without COVID-19.

## Discussion

In this study, more than 8 years of RSV surveillance including 75,128 hospitalized children with ARTI was carried out to better understand the epidemiological characteristics of RSV based on a sentinel hospital in Hubei Province. Yearly RSV epidemic peaks concentrated in the period from December to February were observed before 2021. The RSV detection rate in 2018 and later was significantly higher than that in 2017 and before. RSV was more likely to be detected among younger children and children with an LRTI. The 2-child policy implemented in 2016 was related to an increased RSV detection rate in the following 3 years. The RSV epidemic level decreased sharply from February 2020 to June 2020, but a rebounding epidemic peak in the subsequent winters and increased epidemic levels in the summer of 2021 occurred. The results about the influence of the 2-child policy and COVID-19–related NPIs on the RSV epidemic were obtained using lengthy surveillance data from the Maternal and Child Health Hospital of Hubei Province and will need more multicenter data to further confirm.

RSV was the most frequently detected in the winter and the least frequently detected in the summer in our study, which was similar to the seasonal pattern in some temperate countries [20]. The area in which the Maternal and Child Health Hospital of Hubei Province is located is in a subtropical monsoon climate, with a high temperature in the summer and a low temperature and high relative humidity in the winter. A low temperature is likely to cause vasoconstriction of the nasal mucosa and weakens the immunity of the respiratory tract against viruses [21,22]. In winter, more frequent gathering indoors and a lack of air circulation can also contribute to the transmission of RSV. In addition, relative humidity plays an essential role in respiratory virus transmission. It has been reported that RSV epidemics tend to occur in the rainy season. Wet conditions may increase the number of viruses deposited on surfaces, promote the survival of viruses in droplets on surfaces, and, in turn, encourage the contact transmission of RSV [20,23]. Therefore, prior to rainy or cold weather, knowledge about prevention measures against RSV such as hand hygiene, environmental cleaning, and indoor ventilation should be conveyed to the public, in particular to families with young children.

Limited literature has focused on the longitudinal trends of the RSV epidemic over the past decade in Hubei Province. Most studies devoted to the epidemiological characteristics of RSV in China analyzed the positive rate of RSV among ARTI cases collected during 1 or more years in a cross-sectional manner. A study including pediatric and adult patients with ARTI from 2011 to 2016 in Guangdong Province reported a less clear and unstable seasonal pattern of RSV with epidemics almost year-round [24]. The highest detection rates of RSV usually appeared from March to May and ranged from 15% to 30% in that study. The highest detection rates in that period in our study were similar to those reported in other studies, but RSV broke out earlier. Research conducted in Beijing reported that the peak months of the RSV epidemic were November to January [25]. The results of these studies suggest that earlier RSV epidemics happen with increased latitude and decreased annual average temperatures. Another surveillance study that enrolled 19,898 hospitalized children with ARTI showed an annual RSV detection rate in the range of 5.4% to 7.0% from 2014 to 2018 in China, which was significantly lower than that in our study [26]. Considering the limited sample size and representativeness in that study, we were unable to conclude that the RSV epidemic in our study was more severe than the average countrywide level. More efforts should be taken to improve the surveillance and analyses of the RSV epidemic in different areas in China to formulate tailored prevention measures. Additionally, RSV rates and time trends are totally different across countries. Our study documented an increased RSV detection rate starting in 2018 according to hospital data. In contrast, the RSV infection rate in children with LRTI remained stable, at around 50%, during 2014 to 2018 in the València region in Spain [27,28]. Compared with our results, Germany witnessed similar RSV positivity rates for children aged 0 to 4 years from 2011 to 2021, but the yearly epidemic level was more stable [29]. RSV positivity rates of children fluctuated from 7.5% to 23% during 2007 to 2016 in the Philippines [30]. However, RSV in southwest Finland (2008-2012) and Russia (2013-2018) was rarely detected, with positivity rates of 6% and 4.4%, respectively [31,32]. In general, the area served by the hospital in our study is facing higher pressure from RSV infections in recent years.

The significant increase in the RSV-positive rate since 2018 among young children in our study might be caused by multiple reasons. A potential factor associated with the RSV epidemic among young children was the implementation of the 2-child policy in 2016 in Hubei Province. Since the beginning of the policy, the total number of births rose moderately, while the number of second child births significantly increased [33]. On one hand, the increased number of susceptible infants would contribute to a higher RSV epidemic size. This is in line with a study revealing that there was a sharp increase in inpatient admission rates for infants after China’s universal 2-child policy [34]. In contrast, a modeling study showed that the 1-child policy would lower the average annual influenza attack rate in China [35]. On the other hand, the younger, second child in a family could be infected by other family members, especially their elder siblings. A study in Shanghai elucidated that elder siblings might be the main route of hand, foot, and mouth disease transmission to neonates [36]. Keeping young children from people with flu-like symptoms is a key measure to protect them from RSV infection when a vaccine against RSV is unavailable. In addition, we noted that, although children aged 3 years or older were not directly influenced by the change of the policy after 2017, the RSV epidemic in this age group also increased. There might be 2 reasons. First, RSV is a communicable virus, and the increased scope of RSV infection in infants can also lead to more infections in elder children than before. Second, in addition to the impact of the policy shift, other factors including climate change and more spread of viruses from other provinces could cause an increase in RSV infection in elder children. However, the impact of other factors needs further investigation.

It is universally acknowledged that the COVID-19 pandemic effectively decreased the occurrence of an RSV epidemic due to various measures against the transmission of respiratory viruses [17,37-43]. This phenomenon was even more obvious in the several months under the strictest interventions after the COVID-19 outbreak in our study. However, RSV rebounded to a higher level in the years after strict NPIs were loosened. A delayed outbreak or a severe rebound of RSV was also found in multiple countries [44-48]. NPIs in the context of COVID-19 could prevent the transmission of respiratory viruses but also lead to a greater number of susceptible persons, which might have resulted in larger outbreaks once the transmission routes were reopened [46]. Children aged 1 year to 2 years had a larger rebound than the other age groups in our study. That might be due to the relatively weak immune system and the expanded social activities in this age group. Additionally, the continuous increase in the RSV detection rate among young children with URTI should be noted. Though URTI symptoms are mild, a URTI caused by RSV could progress to LRTI, which has a much higher disease burden [49].

There were some limitations in this study. First, though we used data from a large sample size from the largest maternal and child health hospital in Hubei Province, the conclusions drawn from only 1 hospital might not represent the overall situation at the provincial level. More surveillance studies of RSV from multiple sites are needed to better clarify the epidemiological characteristics of RSV in Hubei Province and perform comparisons among different cities. Genotyping of RSV was not performed; thus, we were unable to elucidate the detailed epidemiological characteristics of different genotypes. Moreover, missing medical records existing for several months in 2016 potentially caused an unprecise estimate of indicators of interest. Nevertheless, despite the limited sample size in February 2016, an epidemic peak was seen, which reflected the stable seasonal pattern of RSV. Only hospitalized children were included in our study, and our findings might overestimate the general level of the RSV epidemic among children. We could only identify the longitudinal trend of RSV and compare the relative levels across different years and different populations. Additionally, preterm birth is a known risk factor for RSV infection [50]. However, due to the lack of this variable in this study, we were unable to further confirm this association or manage the potential confounding effect of preterm birth in the models.

In summary, RSV was frequently detected among hospitalized children with ARTI based on surveillance of data from a large sample size at a hospital in Hubei Province. RSV positivity was more likely to happen in young children, cases diagnosed as LRTI, and wet and low-temperature seasons. Early warning of the risk of RSV infection among high-risk populations and seasons should be considered in local public health practices. A sudden rise in the RSV epidemic after 2018 might be affected by the 2-child policy implementation in 2016. Observed in our study, the RSV epidemic significantly decreased in the short term after the COVID-19 outbreak, followed by a sharp rebound in the years that followed. This implies that healthy lifestyle habits to prevent respiratory viruses should be maintained even though the COVID-19 situation has eased. A specific vaccine against RSV is urgently needed to lower the disease burden attributable to RSV.

## Appendix

Multimedia Appendix 1 Supplementary tables and figures.

Multimedia Appendix 2 Heat maps of respiratory syncytial virus detection rates by week from 2014 to 2022.

Multimedia Appendix 3 Comparison of the respiratory syncytial virus detection rate among periods according to COVID-19 epidemic status (stage 1: February to June in each of 2017, 2018, and 2019; stage 2: February 2020 to June 2020; stage 3: February to June in each of 2021 and 2022).

Multimedia Appendix 4 Comparison of the forecasted respiratory syncytial virus detection rate with the actual status from February 2020 to June 2020 using the seasonal autoregressive integrated moving average.

## Abbreviations

ARTI: acute respiratory tract infection
LRTI: lower respiratory tract infection
NPI: nonpharmaceutical intervention
RR: risk ratio
RSV: respiratory syncytial virus
SARIMA: seasonal autoregressive integrated moving average
URTI: upper respiratory tract infection

## Data Availability

The data sets used and/or analyzed during the current study are available from the corresponding author upon reasonable request.
