# Supplementary material for: Epidemiological Characteristics of Respiratory Syncytial Virus Infection Among Hospitalized Children With Acute Respiratory Tract Infections From 2014 to 2022 in a Hospital in Hubei Province, China: Longitudinal Surveillance Study
Source: JMIR Public Health Surveill. 2023 Apr 27;9:e43941. doi: 10.2196/43941 (PMC10176131; doi:10.2196/43941)
Supplement: Multimedia Appendix 1 [file publichealth_v9i1e43941_app1.docx]

**Supplementary materials**

**Supplementary Table 1. Basic characteristics of samples with URTI**

|  | **Positive/No. of tests** | **Detection rate (95% *CI*), %** | **Adjusted RR (95% *CI*)** | ***P*** |
| --- | --- | --- | --- | --- |
| **Age, years** |  |  |  |  |
| < 1 | 149/5649 | 2.6 (2.3–3.1) | 2.17 (1.43–3.28) | < .001 ^a^ |
| 1–2 | 258/8017 | 3.2 (2.9–3.6) | 2.65 (1.77–3.95) | < .001 ^a^ |
| 3–5 | 170/6181 | 2.8 (2.4–3.2) | 2.26 (1.50–3.40) | < .001 ^a^ |
| 6–17 | 26/2136 | 1.2 0.83–1.8) | Ref. |  |
| **Gender** |  |  |  |  |
| Male | 343/12893 | 2.7 (2.4–3.0) | 0.93 (0.79–1.09) | .37 ^b^ |
| Female | 260/9090 | 2.9 (2.5–3.2) | Ref. |  |
| Note: URTI, upper respiratory tract infection; *CI*, confidence interval; RR, risk ratio; *P*, p–value. ^a^ adjusted for gender; ^b^ adjusted for age. | | | | |

| **Variables** | **Positive/No. of tests** | **Detection rate (95% *CI*), %** | **Adjusted RR (95% *CI*)** | ***P*** |
| --- | --- | --- | --- | --- |
| **Age, years** |  |  |  |  |
| < 1 | 4055/20849 | 19.5 (18.9–20.0) | 13.67 (10.31–18.13) | < .001 ^a^ |
| 1–2 | 2519/15902 | 15.8 (15.3–16.4) | 11.13 (8.38–14.77) | < .001 ^a^ |
| 3–5 | 1111/13026 | 8.5 (8.1–9.0) | 5.98 (4.49–7.97) | < .001 ^a^ |
| 6–17 | 48/3368 | 1.4 (1.1–1.9) | Ref. |  |
| **Gender** |  |  |  |  |
| Male | 4780/32066 | 14.9 (14.5–15.3) | 0.99 (0.95–1.03) | .57 ^b^ |
| Female | 2953/21079 | 14.0 (13.6–14.5) | Ref. |  |
| Note: LRTI, lower respiratory tract infection; *CI*, confidence interval; RR, risk ratio; *P*, p–value. ^a^ adjusted for gender; ^b^ adjusted for age. | | | | |

**Supplementary Table 2. Basic characteristics of samples with LRTI**


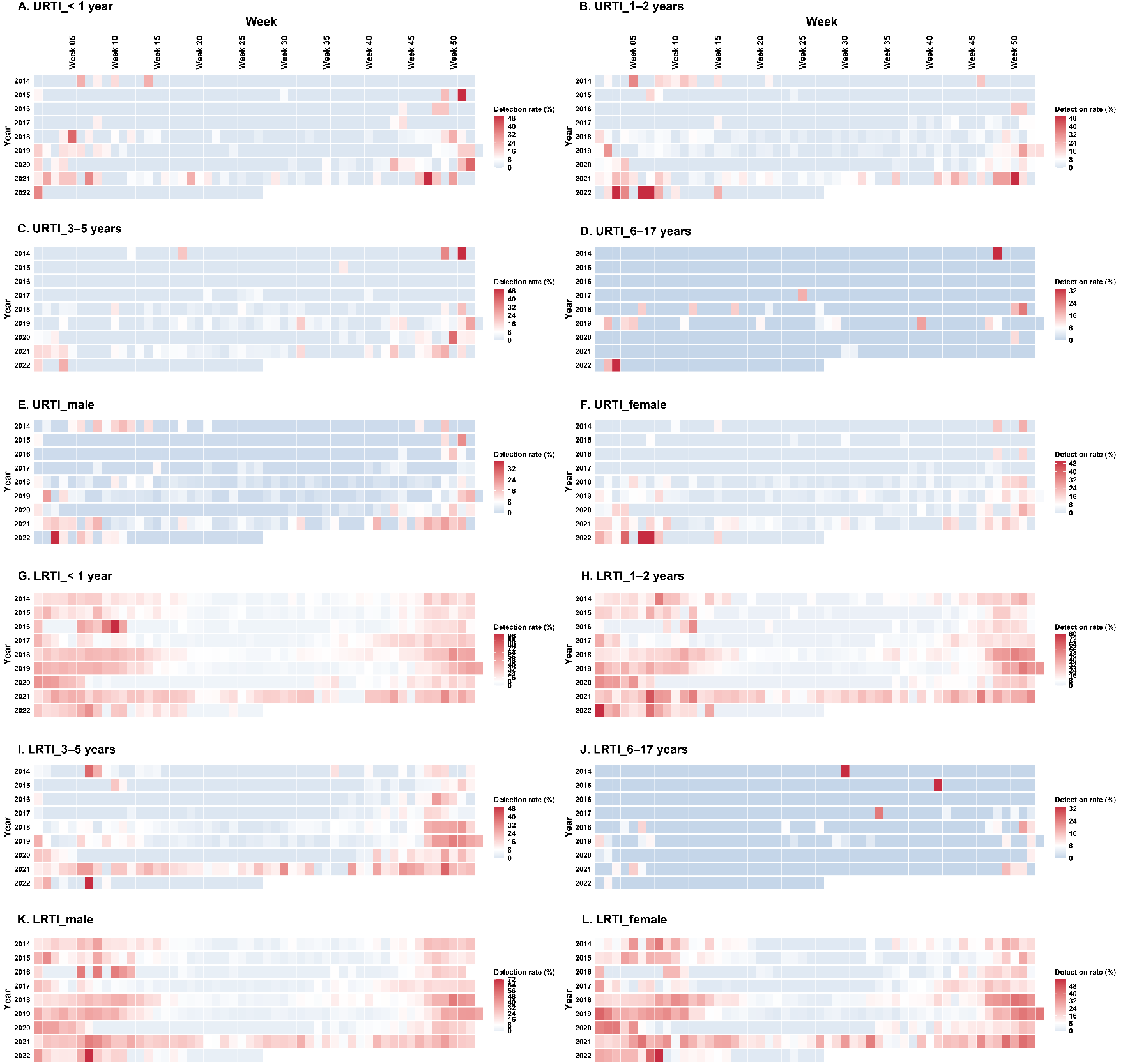


**Supplementary Figure 1. Heatmap of detection rate of respiratory syncytial virus by week from 2014 to 2022**

Note: URTI, upper respiratory tract infection; LRTI, lower respiratory tract infection.


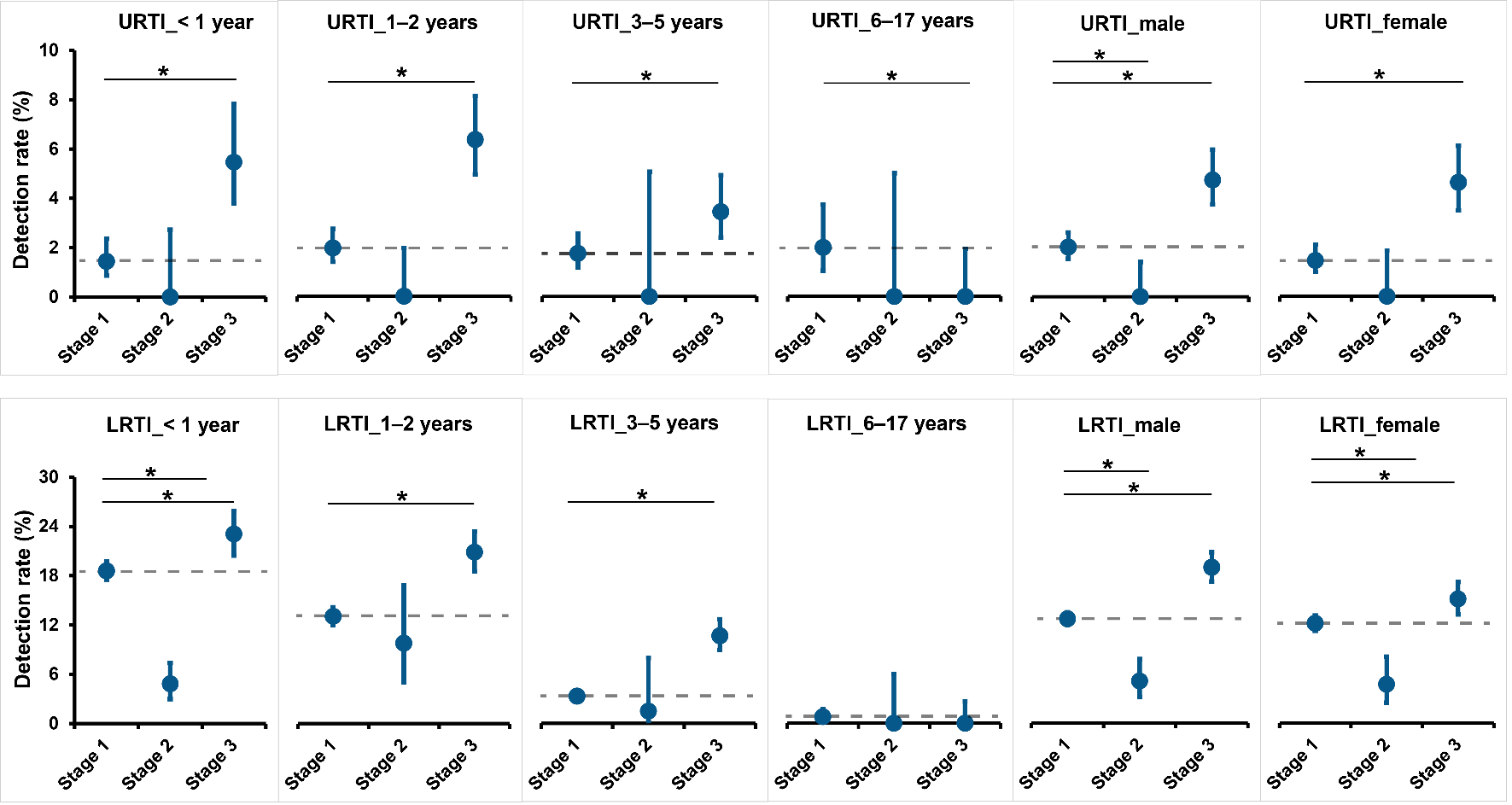


**Supplementary Figure 2. Comparison of the detection rate of respiratory syncytial virus among periods according to COVID-19 epidemic status**

Note: URTI, upper respiratory tract infection; LRTI, lower respiratory tract infection; stage 1, February to June in 2017, 2018, and 2019; stage 2, February to June in 2020; stage 3, February to June in 2021 and 2022.

| **Supplementary Table 3. Comparison of detection rate of respiratory syncytial virus according to the COVID-19 epidemic status** | | | | | | | | | | | | | | |
| --- | --- | --- | --- | --- | --- | --- | --- | --- | --- | --- | --- | --- | --- | --- |
| **Group** | **Stage 1 (2017–2019)** | |  | **Stage 2 (2020)** | |  | **Stage 3 (2021–2022)** | |  | **Stage 2 vs. Stage 1 (ref.)** | |  | **Stage 3 vs. Stage 1 (ref.)** | |
|  | **Positive /No. of tests** | **Detection rate  (95% CI), %** |  | **Positive /No. of tests** | **Detection rate  (95% CI), %** |  | **Positive /No. of tests** | **Detection rate  (95% CI), %** |  | **RD  (95% CI)** | ***P*** |  | **RD  (95% CI)** | ***P*** |
| **Total** | 1600/17010 | 9.41 (8.97–9.85) |  | 32/1135 | 2.82 (1.94–3.96) |  | 680/5744 | 11.84 (11.01–12.7) |  | -6.59 (-7.65–-5.53) | < .001 |  | 2.43 (1.49–3.38) | < .001 |
| < 1 year | 949/6062 | 15.65 (14.75–16.59) |  | 20/550 | 3.64 (2.24–5.56) |  | 249/1456 | 17.10 (15.2–19.14) |  | -12.02 (-13.83–-10.21) | < .001 |  | 1.45 (-0.69–3.59) | .18 |
| 1–2 years | 521/5497 | 9.48 (8.72–10.28) |  | 11/309 | 3.56 (1.79–6.28) |  | 291/2056 | 14.15 (12.67–15.74) |  | -5.92 (-8.12–-3.71) | < .001 |  | 4.68 (2.98–6.37) | < .001 |
| 3–5 years | 115/4200 | 2.74 (2.27–3.28) |  | 1/141 | 0.71 (0.02–3.89) |  | 140/1891 | 7.40 (6.26–8.68) |  | -2.03 (-3.50–-0.56) | .14 |  | 4.67 (3.39–5.94) | < .001 |
| 6–17 years | 15/1251 | 1.20 (0.67–1.97) |  | 0/135 | 0 (0–2.70) |  | 0/341 | 0 (0–1.08) |  | -1.20 (-1.80–-0.60) | .20 |  | -1.20 (-1.80–-0.60) | .04 |
| Male | 977/10143 | 9.63 (9.06–10.22) |  | 20/670 | 2.99 (1.83–4.57) |  | 436/3407 | 12.80 (11.69–13.97) |  | -6.65 (-8.06–-5.24) | < .001 |  | 3.16 (1.9–4.43) | < .001 |
| Female | 623/6866 | 9.07 (8.4–9.78) |  | 12/465 | 2.58 (1.34–4.46) |  | 244/2337 | 10.44 (9.23–11.75) |  | -6.49 (-8.09–-4.9) | < .001 |  | 1.37 (-0.05–2.78) | .051 |
| **URTI** | 82/4659 | 1.76 (1.4–2.18) |  | 0/479 | 0 (0–0.77) |  | 113/2442 | 4.63 (3.83–5.54) |  | -1.76 (-2.14–-1.38) | .003 |  | 2.87 (1.95–3.78) | < .001 |
| < 1 year | 15/1041 | 1.44 (0.81–2.37) |  | 0/137 | 0 (0–2.66) |  | 27/494 | 5.47 (3.63–7.85) |  | -1.44 (-2.16–-0.72) | .16 |  | 4.02 (1.89–6.16) | < .001 |
| 1–2 years | 33/1709 | 1.93 (1.33–2.7) |  | 0/195 | 0 (0–1.87) |  | 58/925 | 6.27 (4.8–8.03) |  | -1.93 (-2.58–-1.28) | .050 |  | 4.34 (2.65–6.03) | < .001 |
| 3–5 years | 25/1450 | 1.72 (1.12–2.53) |  | 0/73 | 0 (0–4.93) |  | 28/826 | 3.39 (2.26–4.86) |  | -1.72 (-2.39–-1.05) | .26 |  | 1.67 (0.26–3.07) | .01 |
| 6–17 years | 9/459 | 1.96 (0.90–3.69) |  | 0/74 | 0 (0–4.86) |  | 0/197 | 0 (0–1.86) |  | -1.96 (-3.23–-0.69) | .22 |  | -1.96 (-3.23–-0.69) | .048 |
| Male | 55/2775 | 1.98 (1.5–2.57) |  | 0/273 | 0 (0–1.34) |  | 67/1434 | 4.67 (3.64–5.9) |  | -1.98 (-2.5–-1.46) | .019 |  | 2.69 (1.48–3.9) | < .001 |
| Female | 27/1884 | 1.43 (0.95–2.08) |  | 0/206 | 0 (0–1.77) |  | 46/1008 | 4.56 (3.36–6.04) |  | -1.43 (-1.97–-0.9) | .08 |  | 3.13 (1.73–4.53) | < .001 |
| **LRTI** | 1518/12351 | 12.29 (11.72–12.88) |  | 32/656 | 4.88 (3.36–6.82) |  | 567/3302 | 17.17 (15.9–18.5) |  | -7.41 (-9.16–-5.67) | < .001 |  | 4.88 (3.47–6.29) | < .001 |
| < 1 year | 934/5021 | 18.6 (17.53–19.71) |  | 20/413 | 4.84 (2.98–7.38) |  | 222/962 | 23.08 (20.45–25.87) |  | -13.76 (-16.09–-11.43) | < .001 |  | 4.48 (1.6–7.35) | .001 |
| 1–2 years | 488/3788 | 12.88 (11.83–13.99) |  | 11/114 | 9.65 (4.92–16.61) |  | 233/1131 | 20.60 (18.28–23.08) |  | -3.23 (-8.76–2.29) | .31 |  | 7.72 (5.13–10.31) | < .001 |
| 3–5 years | 90/2750 | 3.27 (2.64–4.01) |  | 1/68 | 1.47 (0.04–7.92) |  | 112/1065 | 10.52 (8.74–12.52) |  | -1.80 (-4.74–1.14) | .41 |  | 7.24 (5.28–9.20) | < .001 |
| 6–17 years | 6/792 | 0.76 (0.28–1.64) |  | 0/61 | 0 (0–5.87) |  | 0/144 | 0 (e–2.53) |  | -0.76 (-1.36–-0.15) | .50 |  | -0.76 (-1.36–-0.15) | .29 |
| Male | 922/7368 | 12.51 (11.77–13.29) |  | 20/397 | 5.04 (3.1–7.67) |  | 369/1973 | 18.70 (17–20.49) |  | -7.48 (-9.76–-5.2) | < .001 |  | 6.19 (4.31–8.07) | < .001 |
| Female | 596/4982 | 11.96 (11.07–12.9) |  | 12/259 | 4.63 (2.42–7.95) |  | 198/1329 | 14.90 (13.03–16.93) |  | -7.33 (-10.04–-4.62) | < .001 |  | 2.94 (0.82–5.05) | .004 |
| Note: CI, confidence interval; RD, rate difference; *P*, p-value; stage 1, February to June in 2017, 2018, and 2019; stage 2, February to June in 2020; stage 3, February to June in 2021 and 2022. | | | | | | | | | | | | | |  |


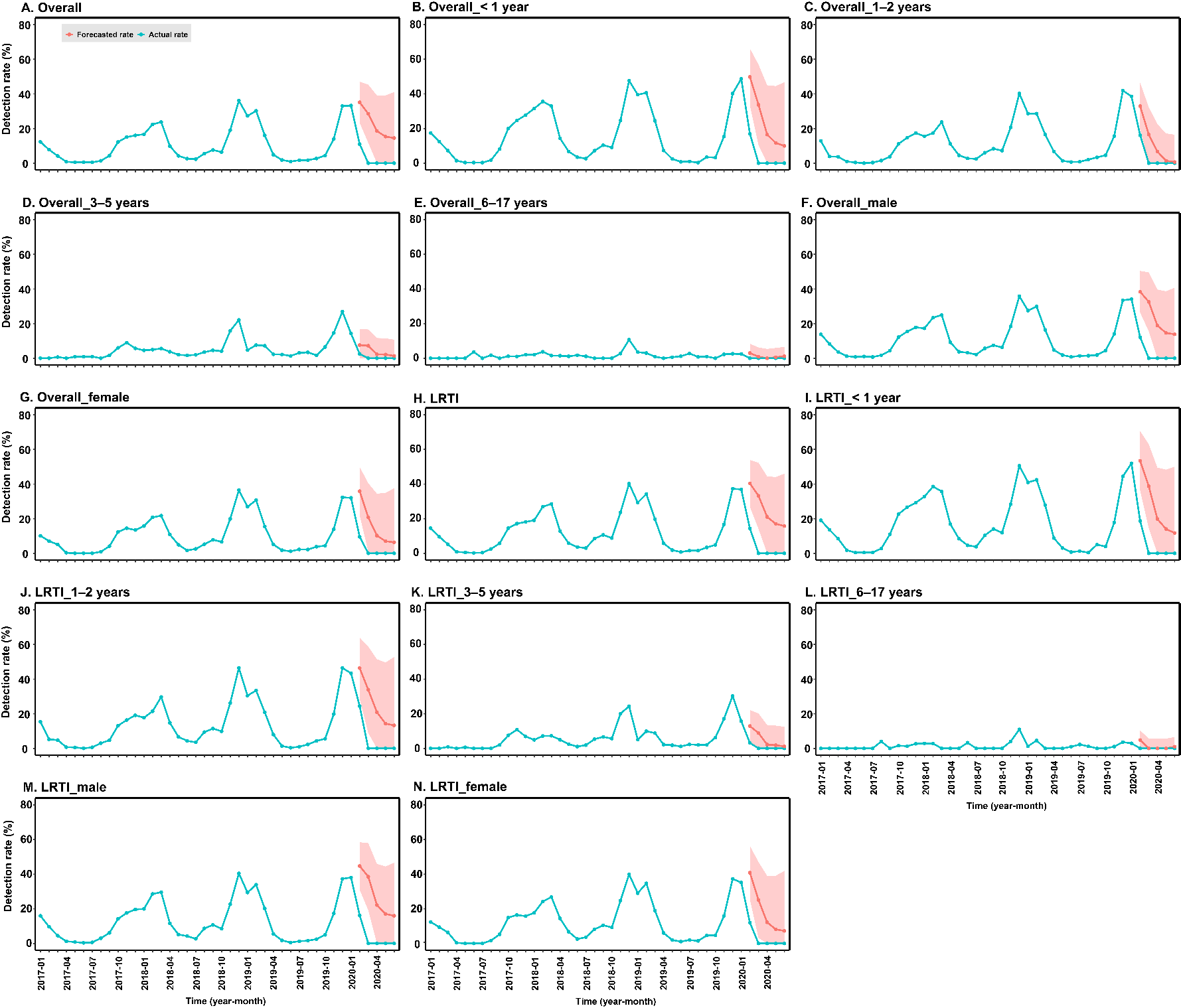


**Supplementary Figure 3. Comparison of forecasted detection rate of respiratory syncytial virus against actual status from February 2020 to June 2020 using** **seasonal autoregressive integrated moving average**
