# Supplementary material for: Epidemiological Characteristics of Respiratory Syncytial Virus Infection Among Hospitalized Children With Acute Respiratory Tract Infections From 2014 to 2022 in a Hospital in Hubei Province, China: Longitudinal Surveillance Study
Source: JMIR Public Health Surveill. 2023 Apr 27;9:e43941. doi: 10.2196/43941 (PMC10176131; doi:10.2196/43941)
Supplement: Multimedia Appendix 2 [file publichealth_v9i1e43941_app2.docx]

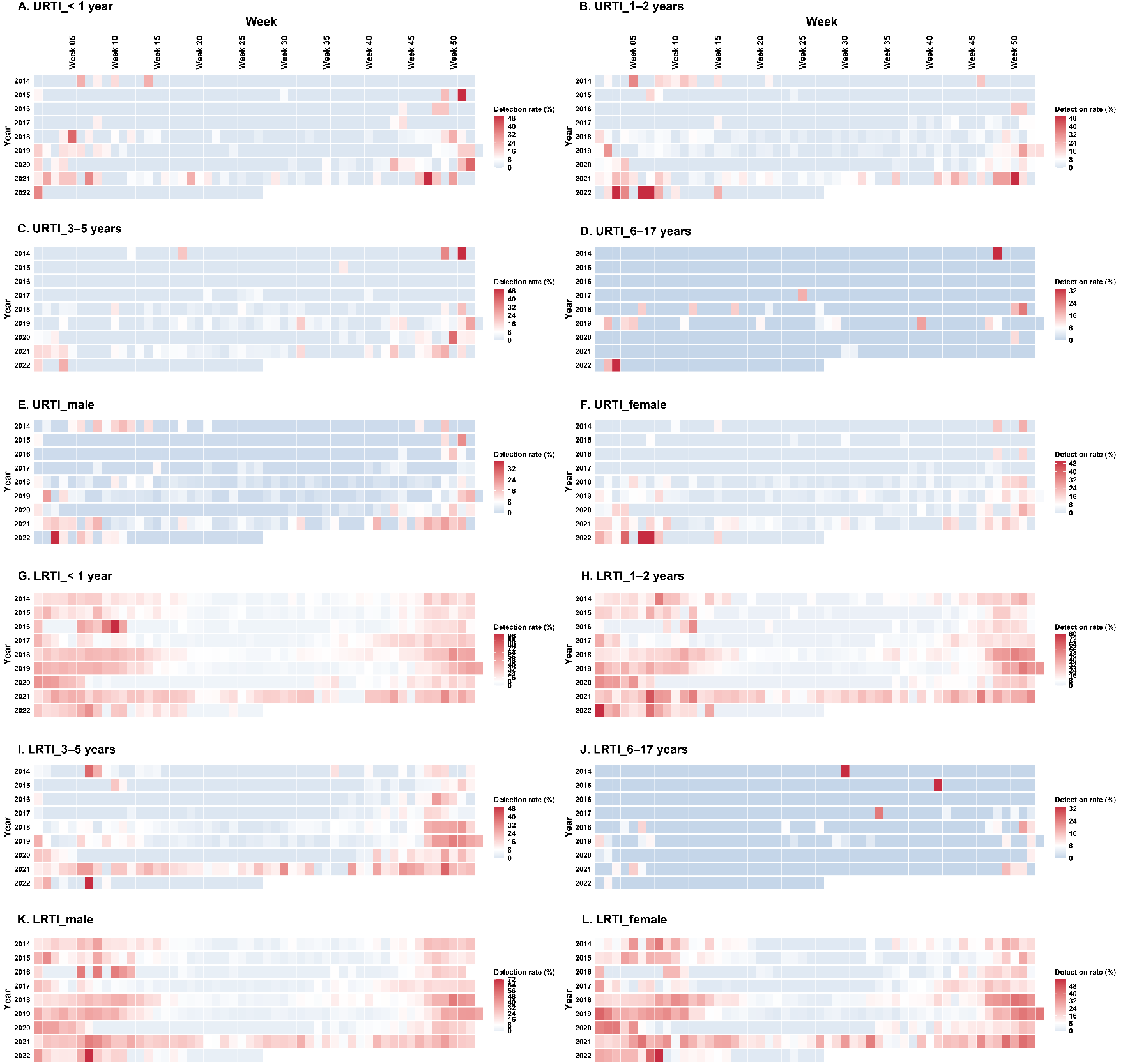
**Figure S1.** Heat maps of respiratory syncytial virus detection rates by week from 2014 to 2022. LRTI: lower respiratory tract infection; URTI: upper respiratory tract infection.
