# Supplementary material for: Epidemiological Characteristics of Respiratory Syncytial Virus Infection Among Hospitalized Children With Acute Respiratory Tract Infections From 2014 to 2022 in a Hospital in Hubei Province, China: Longitudinal Surveillance Study
Source: JMIR Public Health Surveill. 2023 Apr 27;9:e43941. doi: 10.2196/43941 (PMC10176131; doi:10.2196/43941)

**Figure S2.** Comparison of the respiratory syncytial virus detection rate among periods according to COVID-19 epidemic status (stage 1: February to June in each of 2017, 2018, and 2019; stage 2: February 2020 to June 2020; stage 3: February to June in each of 2021 and 2022). LRTI: lower respiratory tract infection; URTI, upper respiratory tract infection.


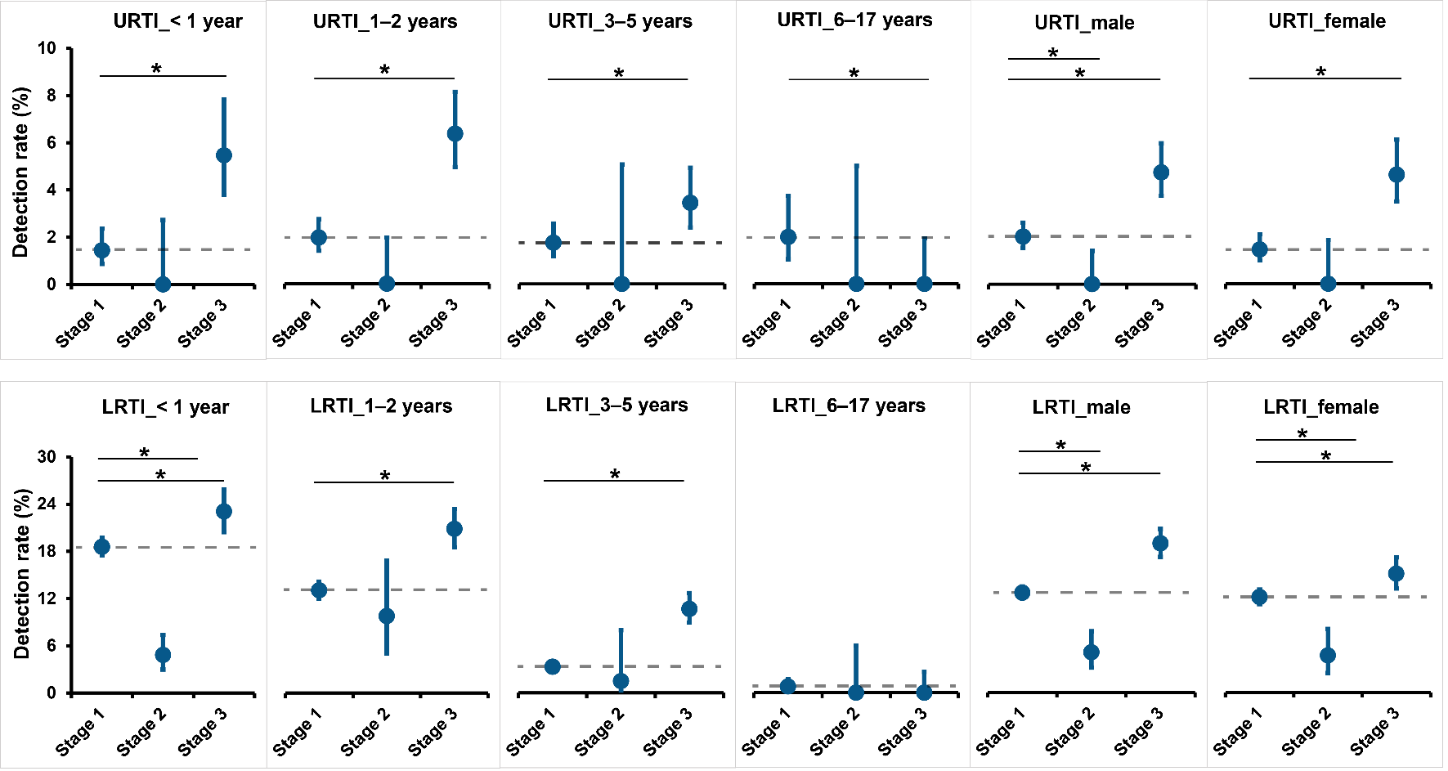

Supplement: Multimedia Appendix 3 [file publichealth_v9i1e43941_app3.docx]
