# Supplementary material for: Epidemiological Characteristics of Respiratory Syncytial Virus Infection Among Hospitalized Children With Acute Respiratory Tract Infections From 2014 to 2022 in a Hospital in Hubei Province, China: Longitudinal Surveillance Study
Source: JMIR Public Health Surveill. 2023 Apr 27;9:e43941. doi: 10.2196/43941 (PMC10176131; doi:10.2196/43941)

**Figure S3.** Comparison of the forecasted respiratory syncytial virus detection rate with the actual status from February 2020 to June 2020 using the seasonal autoregressive integrated moving average.


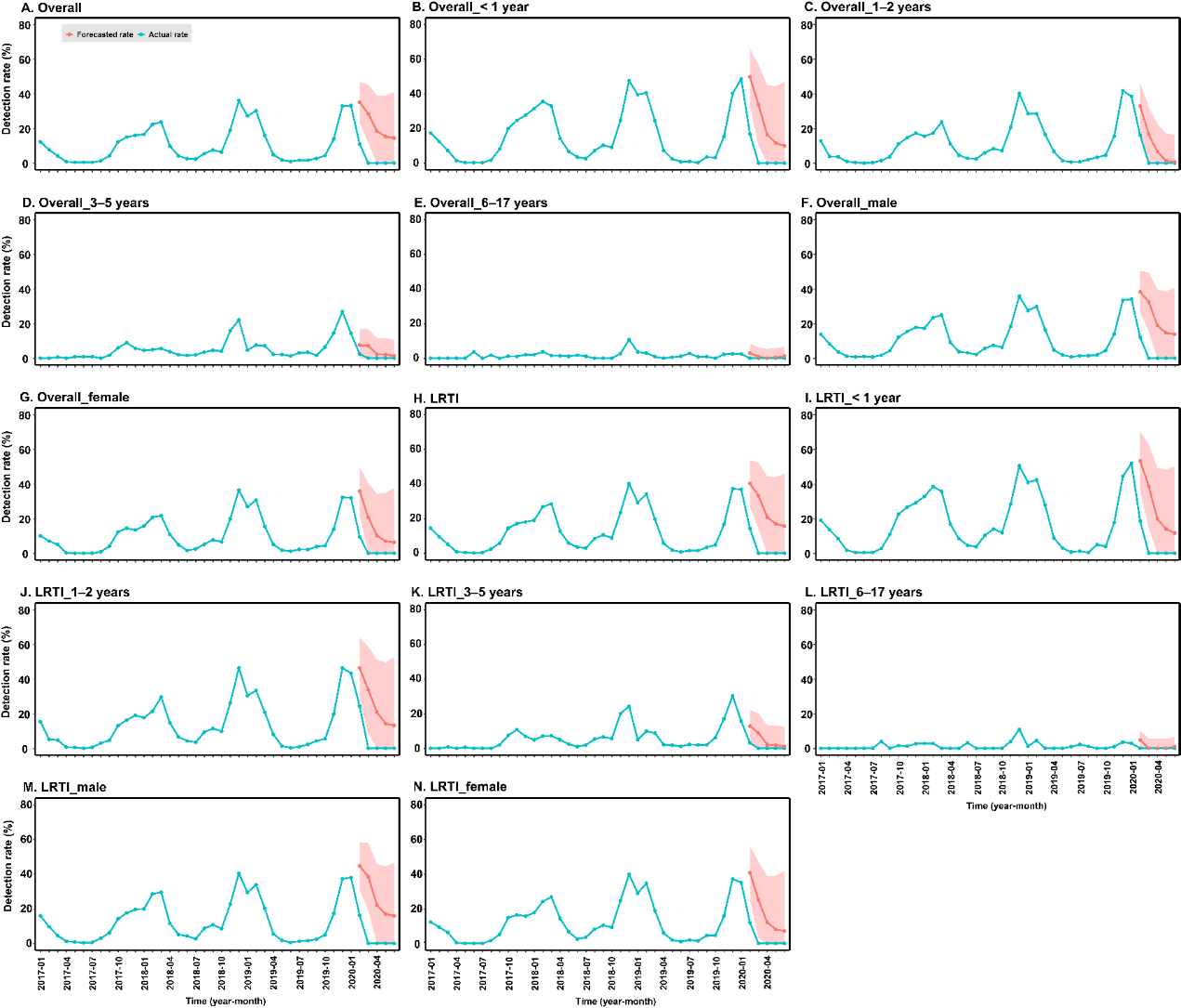

Supplement: Multimedia Appendix 4 [file publichealth_v9i1e43941_app4.docx]
